# The Great Cold Spot in Jupiter's upper atmosphere

**DOI:** 10.1002/2016GL071956

**Published:** 2017-04-10

**Authors:** Tom S. Stallard, Henrik Melin, Steve Miller, Luke Moore, James O'Donoghue, John E. P. Connerney, Takehiko Satoh, Robert A. West, Jeffrey P. Thayer, Vicki W. Hsu, Rosie E. Johnson

**Affiliations:** ^1^ Department of Physics and AstronomyUniversity of LeicesterLeicesterUK; ^2^ Department of Physics and AstronomyUniversity College LondonLondonUK; ^3^ Center for Space PhysicsBoston UniversityBostonMassachusettsUSA; ^4^ Goddard Space Flight CenterNASAGreenbeltMarylandUSA; ^5^ Institute of Space and Astronautical ScienceJAXASagamiharaJapan; ^6^ Jet Propulsion LaboratoryCalifornia Institute of TechnologyPasadenaCaliforniaUSA; ^7^ Aerospace Engineering SciencesUniversity of Colorado BoulderBoulderColoradoUSA

**Keywords:** Jupiter, thermosphere, aurora, ionosphere, infrared, vortex

## Abstract

Past observations and modeling of Jupiter's thermosphere have, due to their limited resolution, suggested that heat generated by the aurora near the poles results in a smooth thermal gradient away from these aurorae, indicating a quiescent and diffuse flow of energy within the subauroral thermosphere. Here we discuss Very Large Telescope‐Cryogenic High‐Resolution IR Echelle Spectrometer observations that reveal a small‐scale localized cooling of ~200 K within the nonauroral thermosphere. Using Infrared Telescope Facility NSFCam images, this feature is revealed to be quasi‐stable over at least a 15 year period, fixed in magnetic latitude and longitude. The size and shape of this “Great Cold Spot” vary significantly with time, strongly suggesting that it is produced by an aurorally generated weather system: the first direct evidence of a long‐term thermospheric vortex in the solar system. We discuss the implications of this spot, comparing it with short‐term temperature and density variations at Earth.

## Key Points

- The detection of a Great Cold Spot in Jupiter's upper atmosphere, which is quasi‐stable over a 15 year period
- The Great Cold Spot is approximately fixed in magnetic latitude and longitude but changes in shape and size on moderate timescales
- We interpret this as direct evidence of the first long‐term thermospheric vortex in the solar system: an aurorally generated weather system

## 1 Introduction

The upper atmosphere of a planet represents an important boundary region between the underlying atmosphere and the surrounding space environment, consisting of the coexisting neutral thermosphere and charged ionosphere. The thermosphere is dominated by inputs from the atmosphere below, heating from the Sun, and, for magnetized planets, indirectly through currents that form between the ionosphere and the surrounding magnetosphere.

At Earth, the global thermospheric temperature varies widely, between 600 and 2000 K, as a result of changing geomagnetic activity and solar EUV flux [*Roble*, 1983]. During periods of low geomagnetic activity, the thermosphere is dominated by atmospheric waves, with the dayside of the planet being heated by EUV from the Sun, and additional significant contributions propagating upward as waves from major disturbances within the underlying stratosphere [*Vincent*, 2015]. However, during periods of enhanced geomagnetic activity, coupling between the ionosphere and the surrounding magnetosphere results in significant heating of the thermosphere in the polar regions, as well as strong momentum coupling with antisunward ion winds across the poles. This high‐latitude heating and momentum transfer results in a “surge” of gravitational waves that propagates to the equator and beyond, redistributing the auroral energy across the entire thermosphere [*Forbes*, 2007].

Jupiter's thermosphere is quite unlike the Earth's. The equatorial thermospheric temperature ranges from <700 up to 1200 K [*Lam et al.*, 1997; *Seiff et al.*, 1997], while modeling of the expected solar heating of Jupiter's thermosphere predicts equatorial temperatures of only ~200 K [*Yelle and Miller*, 2004]. However, unlike Earth, some Jovian thermospheric models have shown that while enhanced jovimagnetic activity can result in dramatic heating within the polar regions [*Melin et al.*, 2006], this heat cannot be transported from the poles to equator due to Coriolis forces [*Achilleos et al.*, 1998; *Smith and Aylward*, 2009; *Yates et al.*, 2012], though other models disagree, showing auroral flows converging and downwelling at the equator [*Majeed et al.*, 2005]. The rapid rotation of Jupiter, combined with its large size, results in very strong Coriolis forces which quickly bend meridional winds flowing toward the equator into zonal winds around the planet, so that even very high jovimagnetic activity can only heat the equatorial region by ~50 K [*Yates et al.*, 2014]. The high equatorial temperatures may well originate locally, formed by heat propagating upward from lower altitudes, with recent observations suggesting very large thermospheric heating associated with the Great Red Spot [*O'Donoghue et al.*, 2016]. Differing temperatures could also result from different ionization processes, with the peak auroral ionization occurring deeper within the atmosphere than the peak solar EUV ionization. This change in the peak ionization altitude could mean that H_3_^+^ at the equator is thermalized in a higher‐altitude, hotter thermosphere than the aurora [*Tao et al.*, 2011].

The result of the trapping of auroral energy at jovigraphic polar latitudes means that the thermosphere has a thermal gradient between the auroral region and midlatitudes, ranging between >1000 K and <700 K, respectively [*Lam et al.*, 1997].

The aurorae of Jupiter, generated by a variety of interactions with the surrounding magnetosphere, result in a variety of complex emissions [*Badman et al.*, 2015]. Because of the tilt in Jupiter's magnetic dipole, the northern aurora drapes down to jovigraphic latitudes as low as 56°N at a longitude of 180°W but only reaches as low as 88°N latitude at 0°W [*Grodent et al.*, 2008]. The southern aurorae are more circular in shape, varying between 68°S and 84°S and thus laying closer to the rotational pole.

However, measurements of the thermalized ionic molecule H_3_^+^, using data taken during the Cassini flyby of Jupiter, show that no H_3_^+^ emission is found on Jupiter's nightside at magnetic latitudes below those mapping to Io [*Stallard et al.*, 2015], suggesting that H_3_^+^ emission measured on the dayside below these latitudes is entirely generated by solar EUV ionization. In the past, this “mid‐to‐low”‐latitude dayside emission has been shown to reduce gently with latitude down to a minimum at the equator [*Rego et al.*, 2000; *Stallard et al.*, 2012], with enhanced subauroral emission extending along lines of jovigraphic longitude away from the auroral region [*Miller et al.*, 1997; *Morioka et al.*, 2004]. This gentle gradation in emission is suggestive of a diffuse thermal gradient away from Jupiter's dynamic aurora. Indeed, current models of the coupled ionosphere‐thermosphere system predict this smooth temperature gradient away from the auroral region [*Achilleos et al.*, 1998; *Bougher et al.*, 2005].

In this paper, we describe new observations that reveal unexpected localized variations in Jupiter's subauroral thermosphere, suggesting that this region is dominated by complex dynamic flows and quasi‐stable vortices: aurorally generated weather systems within Jupiter's thermosphere.

## 2 H_3_^+^ Spectral Images

Observations of the auroral region of Jupiter were taken using the Cryogenic High‐Resolution IR Echelle Spectrometer (CRIRES) instrument [*Käufl et al.*, 2004] on the Very Large Telescope (VLT) on 17 October and 31 December 2012. These observations consisted of a sequence of long‐slit spectra, with the slit aligned east‐west on the planet, and scanned in a repeating sequence from the planetary limb down through the entire auroral region in steps of 0.1″. These data were flat fielded, straightened using sky lines, and flux calibrated using light from an A0V star, in the usual manner. The instrument observed a wavelength range between 3.885 and 3.986 µm, accessing several emission lines from the H_3_^+^ fundamental Q branch within the ionosphere. This is also a spectral region in which there is significant hydrocarbon absorption by the underlying, immediately lower atmosphere, which effectively removes any reflected sunlight from the spectra.

**Figure 1:**
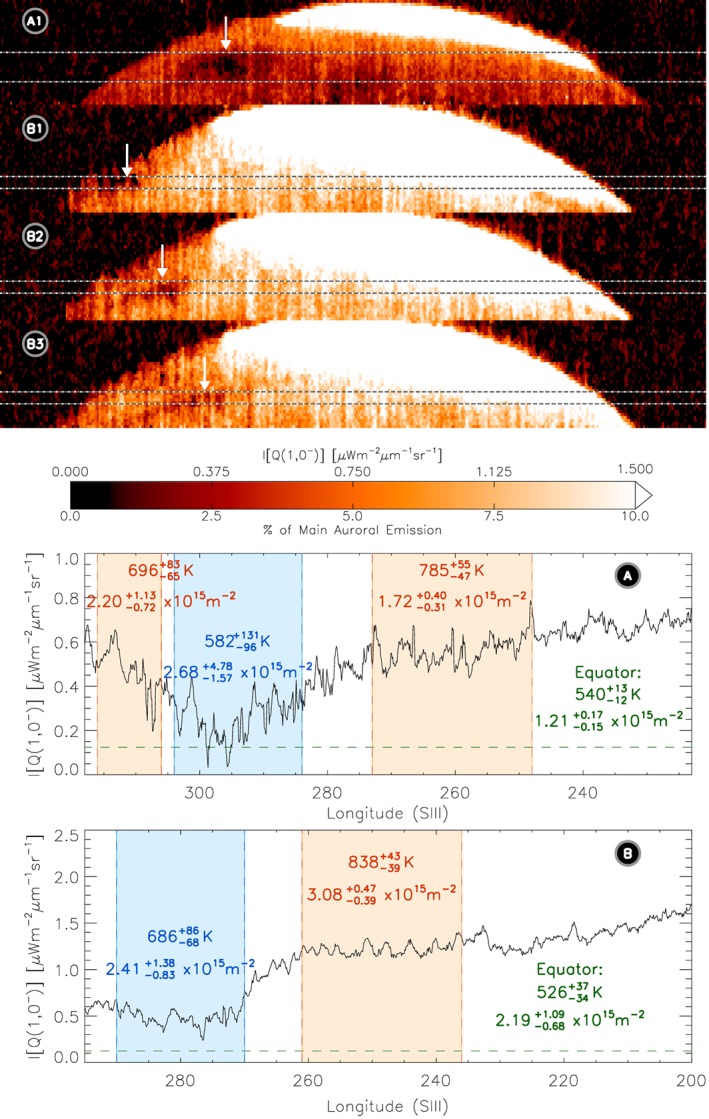
H_3_^+^ emission measured by the CRIRES on VLT. These observations were made on (a) 17 October and (b) 31 December 2012. Images of emission from the H_3_^+^ ν2 Q(1,0^−^) line, top, have been scaled to highlight subauroral emission, as shown by the color bar, and clearly show a region of darkening in the ionosphere (highlighted with arrows). The H_3_^+^ emission from these images has been summed over the latitudinal region of the darkening (demarked within the images by horizontal dotted lines), to produce two plots of varying H_3_^+^ emission with system III longitude, bottom, one for 17 October and one for 31 December. Here the region of darkening can be clearly seen for both nights. We have calculated the mean temperature and column density, the two values displayed within each region, both inside the dark region (blue), and in the surrounding ionospheric regions (red). Using separate data, we have also calculated the mean equatorial emissions (dashed line), temperature, and column density for each night, measured within an hour of these observations (green).

Combining the emission from the H_3_^+^ lines allowed a series of spectral maps to be produced, observing the aurora as the planet rotated, with line brightness measured using established Gaussian fitting measurement techniques [e.g., *Stallard et al.*, 2002]. Although the observations were intended to measure conditions within the auroral/polar region, a combination of the light collecting power from the 8 m VLT and the sensitivity of the CRIRES instrument also allowed us an unprecedented view of the subauroral regions, as show in Figure 1.

The main auroral emission brightness, the H_3_^+^ ν2 Q(1,0^−^) line on 31 December, was approximately 15 μW^−2^ µm^−1^ sr^−1^. Scaling the brightness of the images to 10% of this value, we note what appears to be a highly unexpected localized region of darkening, well away from the main emission, highlighted with arrows in Figure 1. This feature occurs on both 17 October and 31 December and appears to corotate with the planet on 31 December. However, while the feature occurs at a latitude ~55°N, it has clearly moved in longitude, with its center shifting from 300°W to 270°W.

Because H_3_^+^ is in quasi‐thermal equilibrium in Jupiter's ionosphere [*Miller et al.*, 1990], variations in emission can be driven by either changes in the local H_3_^+^ density or by changes in temperature within the surrounding neutral atmosphere. We can calculate temperature and column density by measuring and comparing the H_3_^+^ emission from two separate emission lines [e.g., *Stallard et al.*, 2002; *Melin et al.*, 2014]. We use emission from the H_3_^+^ ν2 Q(1,0^−^) line at 3.9530 µm, and the H_3_^+^ ν2 Q(3,0^−^) line at 3.9855 µm.

Even using VLT/CRIRES, the H_3_^+^ emission strength is too weak to accurately calculate the temperature and column density on a pixel‐by‐pixel basis. Instead, we designate regions from our observations: one focused on emission from the dark region itself and others on emission from longitudes east and west of the dark region. The latitudinal range covered is shown in Figure 1 as horizontal dashed lines. From these, we calculate the mean H_3_^+^ intensity with longitude, a plot shown in Figures 1a and 1b. By comparing the emission from the two H_3_^+^ lines across each region, we measure the mean temperature and column density in each.

Using this, we clearly show that the dark region is significantly cooler than the surrounding regions, with temperatures separated by more than their combined errors, and with calculated temperatures inside the dark region on the order of 200 K cooler than the surrounding regions, though it is notably still warmer than the equatorial region. Such temperature changes can completely explain the significant drop in H_3_^+^ emission within the dark region. In contrast with this, the column densities in both the dark region and the surrounding ionosphere appear to be similar, though the errors associated with the calculated column densities are too large to be certain that there is not also a significant change in density within this region. It is possible that this apparent cooling is the result of a localized breakdown in quasi‐thermal equilibrium. However, for such a change to occur, it requires a significant change in the altitude of peak ionization, and given that all the H_3_^+^ observed in Figures 1a and 1b is likely generated by solar EUV ionization, such a localized change in the ionization is difficult to explain.

Assuming that the H_3_^+^ is in quasi‐thermal equilibrium with the surrounding neutrals, this suggests a localized thermospheric feature which covers a region of ~30° of longitude and ~20° of latitude, close in scale to the Great Red Spot. For simplicity, we will refer to this region of cooling as the Great Cold Spot (GCS) for the rest of this paper.

## 3 Average H_3_^+^ Emission

**Figure 2:**
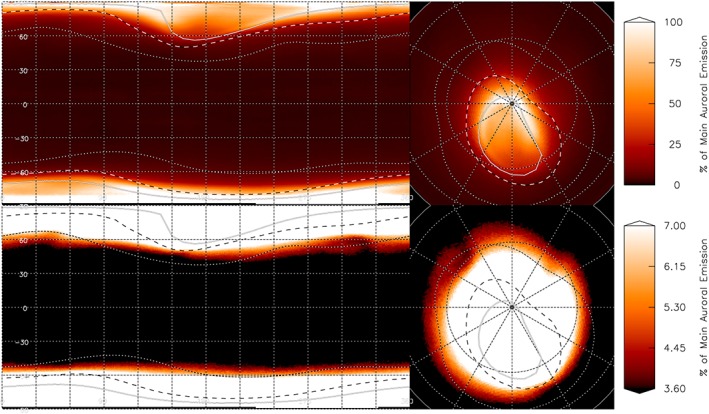
A map of mean H_3_^+^ emission across the 5 year Connerney and Satoh H_3_^+^ Jupiter image database. A latitude/longitude orthographic projection is shown on the left and a polar projection on the right. All maps show the emission scaled against the peak auroral brightness at latitudes between 75°N and 75°S with a gamma stretch of 0.5, and the bottom maps show a narrow range of emission brightness, scaled to highlight the subauroral region, as shown by the color bar. We identify the ionospheric locations that magnetically map to the main auroral emission (solid line; 30 *R_J_* and Io spot and tail (dashed‐line; 5.9 *R_J_*) [*Grodent et al*., 2008], and to Amalthea (dotted line; 2.544 *R_J_*) [*Connerney et al*., 1998]. Also shown are the jovigraphic longitude and latitude in 30° steps (grey dotted line).

In order to better understand the dynamics of the feature, we need to identify and characterize the GCS over a much longer time frame. During the period between 1995 and 2000, J. Connerney and T. Satoh took extensive images of H_3_^+^ emission from Jupiter's ionosphere [e.g., *Satoh et al.*, 1996], using the NSFCam instrument on NASA's InfraRed Telescope Facility [*Shure et al.*, 1994]. This data set was recently reprocessed by M. Lystrup and B. Bonfond for the “Magnetospheres of the Outer Planets Infrared Data Archive.” We have taken 13,501 images observed by Connerney and Satoh, across 48 nights of observation, using a range of filters that cover the 3.4–3.6 µm range. These images were then processed to remove ghosting, projected into latitude and longitude maps, and added together across the entire 6 year period, using the techniques described in the supporting information. (Details of the instrument settings and techniques used are also described in the supporting information). The resultant map of time‐averaged H_3_^+^ emission from Jupiter's ionosphere is shown in Figure 2.

Given the range of instrument settings used over the 5 year period, an absolute intensity calibration is not necessarily meaningful. Instead, we have scaled our data with respect to the peak auroral brightness at latitudes equatorward of 75°N and 75°S (in order to minimize the effects of line‐of‐sight brightening). Although the region in question is weaker than the main auroral emission, once the map is scaled to highlight the subauroral ionosphere, a region of ionospheric darkening can clearly be seen in the data, centered on 55°N and 300°W. This shows that the cold spot observed in Figure 1 is a persistent feature of thermospheric cooling fixed in system III longitude over a period of more than 15 years. A possible second region of dimming can also be seen around 60°N and 40°W. It is also notable that when scaled in this way, the weakest emission no longer follows the outline of the main auroral oval but instead appears to broadly follow lines of jovigraphic latitude.

**Figure 3:**
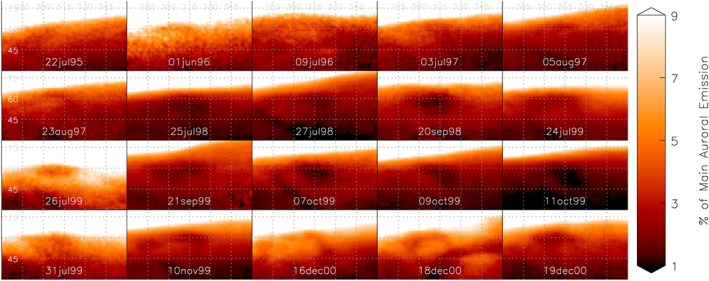
A clipped region of the latitude/longitude map (30–80°N, 270–360°W), showing how emission from this region varies with time over the 1995–2000 period. Emission is scaled from the peak auroral brightness at latitudes between 75°N and 75°S with a gamma stretch of 0.5, to highlight the subauroral region, as shown in the color bar. Each frame has been smoothed by 2° of latitude and longitude, in order to improve the signal to noise ratio, and lines of 15° of latitude and longitude are shown (grey dotted line). Between 1995 and 1997, there are significant variations in this region, but no clear spot, and from 1998 onward a clear dark region occurs within each of these images, evolving in location and morphology.

In Figure 3, we show a localized segment of the H_3_^+^ latitude and longitude map of the ionosphere (30–80°N, 270–360°W) from multiple nights across the 5 year Connerney and Satoh data set. In early years, between 1995 and 1997, although there is localized variability, no clear spot can be seen. However, between 1997 and 1998 the GCS can be seen to form. The shape and location of the GCS then evolve with time to 2000; it is not strongly fixed in either latitude or longitude and is often ringed by a region of emission brighter than the surrounding ionosphere. The morphology of the dark region also sometimes appears considerably distorted, but it typically forms a roughly oval shape. This suggests that the cold spot is a quasi‐stable feature, in that on shorter timescales the spot can drift in system III latitude and longitude by 10–20°, and sometimes significantly change in morphology, but statistically will always reoccur at the same approximate location on the planet.

## 4 Discussion

The detection of a localized region of cooling within the upper atmosphere is unexpected. Current models and previous observations have both been limited by spatial resolution, so that they are relatively insensitive to finer‐scale structures. As a result, they have suggested that there are variations within the thermosphere that may occur on relatively large scales and that thermospheric energy flows should act to smooth out and limit more local temperature variations. Indeed, even within the auroral region, where the thermosphere may be heated by very localized energy sources, the temperatures typically vary by only 100–200 K [*Stallard et al.*, 2002; *Raynaud et al.*, 2004]. In contrast, our latest study suggests that the GCS represents either a highly localized region of cooling or that strong dynamics disturb the equilibrium.

One possible driver for localized dynamics could be an underlying weather system propagating localized winds from the lower stratosphere or troposphere. However, such weather systems typically result from flow shears within the lower atmosphere and as a result move relative to the system III longitude on a yearly timescale; in addition, low‐altitude weather could only provide energy into the thermosphere though wave interaction, and it is difficult to envisage a way that this could drive localized cooling. Past observations of Jupiter's stratosphere reveal a localized hot spot focused on the auroral region near system III longitude 180 [*Livengood et al.*, 1993]. This hot spot within the northern auroral region is apparently associated with the occasional formation of a large dark oval seen at near‐UV wavelengths [*Porco et al.*, 2003; *West et al.*, 2004]. However, away from the auroral region, stratospheric UV structure, mid‐IR hydrocarbon thermal emission, and temperatures all vary smoothly along lines of equal latitude, with no evidence of localized variability near the location of the Great Cold Spot [*Porco et al.*, 2003; *West et al.*, 2004; *Flasar et al.*, 2004]. This suggests that the atmospheric cooling observed here is confined to the thermosphere alone, above the stratosphere.

Since the Great Cold Spot is effectively fixed in magnetic longitude, a second potential source for the cool region could be interactions with the surrounding magnetosphere. However, it is typically difficult to produce auroral darkening, or localized cooling, via auroral processes. It could be that this darkening represents a region in which H_3_^+^ is destroyed by a drizzle of infalling water ions via pitch angle scattering—a Jovian equivalent of the “Ring Rain” seen on Saturn [*O'Donoghue et al.*, 2013]. However, the cold spot occurs well away from the region where the peak pitch angle scattering is thought to reach a maximum, estimated at magnetic latitudes mapping to 1.5 *R_J_* and at longitudes of 30°W [*Abel and Thorne*, 2003]. The GCS maps into the magnetosphere slightly farther out than Amalthea, at 2.54 *R_J_*, where there is no obvious source of plasma to produce such precipitation. In addition, interactions with the magnetosphere cannot explain the changes in longitude observed over longer timescales. Given that the GCS is observed over an extended period of time, any magnetospheric source would have to be fixed in system III longitude and long lived, or the affect of the source would be averaged out across all system III longitudes. Unless the magnetospheric source originated well away from the magnetospheric equatorial plane, it is also likely that it would produce a magnetically conjugate cool dark region in the southern hemisphere; no such feature is observed in Figure 2.

The low temperatures within the GCS might be driven by a change in the peak H_3_^+^ ionization altitude. However, for this to produce a cooling, it suggests ionization either deeper in the atmosphere, resulting in a cooler surrounding thermosphere, or high enough in the atmosphere that quasi‐thermal equilibrium breaks down. This would again require a localized auroral ionization process, as solar EUV ionization is broadly uniform across the Jovian disk.

Since this feature is unlikely to be driven by the atmosphere below the ionosphere, or by direct interactions with the magnetosphere, this suggests that the GCS originated from processes that occur within, or at close altitudes to, the thermosphere. The observed cooling might be driven by thermospheric dynamics. However, since the H_3_^+^ in this region is likely produced through solar EUV ionization, the changing morphology seen over moderate timescales also strongly implies that the GCS is created by dynamic interactions within the upper atmosphere, which would be expected to vary on such timescales.

At Earth, ion drag, Coriolis, centrifugal, and pressure‐gradient forces are the primary forces in the thermosphere with their relative magnitudes influenced by heating in the auroral region and plasma convection. The combination of these forces can lead to sustained vortex structures in the thermosphere that induce secondary circulations resulting in adiabatic cooling of the vortex core creating cold spots [*Walterscheid and Crowley*, 2009]. Thermosphere model simulations and some supporting observations indicate the presence of vortices with cold centers and that these vortices and associated temperatures will vary with geomagnetic activity [e.g., *Schoendorf and Crowley*, 1995]. Modeling shows that such vortices would occur at subauroral latitudes and at longitudes away from the location of the main auroral energy deposition and are dynamically forced, either as instabilities produced on the edge of the flows [*Walterscheid and Crowley*, 2009] or nonlinear dynamic imbalances driven by the flows [*Walterscheid and Crowley*, 2015]. The imbalance of flows for a vortex can result in a cold center depending on the direction of rotation and the relative magnitudes of Coriolis, centrifugal, and pressure gradients. As explained in *Walterscheid and Crowley* [2015], a cyclonic thermosphere vortex will produce a cold center when Coriolis is stronger than centrifugal forces, meaning relatively low wind speeds and modest radius of curvature in the wind vortex. Anticyclonic circulations can also produce cold cores when the centrifugal forces are stronger than Coriolis and largely occur when strongly forced by geomagnetic activity.

The jovigraphic latitude of this feature, occurring near 55°N, is close to the equatorward limit of the northern main auroral emission. As we have seen, models predict that auroral energy deposited into the thermosphere is likely to migrate around the planet along lines of equal jovigraphic longitude. In addition, plasma convection within the auroral region, which drives strong neutral flows at the Earth, could also have a significant contributory factor at Jupiter, as indicated by past models of ionosphere‐thermosphere interactions [*Achilleos et al.*, 1998; *Bougher et al.*, 2005; *Millward et al.*, 2005; *Majeed et al.*, 2016]. The strongest of these flows has been shown to result within polar regions that interact with the solar wind, where ions are held “stationary” in the inertial frame producing a very strong subrotational flow in the planetary frame [*Stallard et al.*, 2001]. These processes would result in bulk flows within the polar thermosphere, contained at jovigraphic latitudes through which the northern aurora extends; the GCS is located at the boundary between this aurorally enhanced polar thermosphere and the more quiescent equatorial region, as highlighted in Figure 2. Within Jupiter's lower atmosphere, boundary regions between two regions of bulk flow with different energies often form relatively small and stable vortexes [*Ingersoll*, 1990].

Similar to the sustained vortices in Earth's thermosphere, the GCS appears to be quasi‐stable. The majority of Jupiter's aurorae are produced as a result of continuously driven internal current systems, driven by plasma from Io, with regions of auroral heating fixed at the same jovigraphic latitudes and longitudes. This would result in a positionally stable flow of energy away from this region, so that over a 15 year period, the weather system produced in response to the flow of this energy must also remain somewhat stable. A matching region of cooling is not observed in the southern hemisphere, as here the auroral region is more symmetrically distributed around the rotational pole, positioning any thermospheric cooling close to the auroral region itself.

However, the intensity of Jupiter's aurora can change significantly as a result of changes in the solar wind [*Baron et al.*, 1996], Io's volcanic activity [*Kimura et al.*, 2015], or other unknown processes [*Badman et al.*, 2016]. As such, the energy transport will change on shorter timescales, explaining the longitudinal quasi‐stability of the GCS, with significant changes in position and morphology within individual observations and with the spot sometimes not observed at all. This long‐term quasi‐stability suggests that like Jupiter's UV Great Dark Spot [*Porco et al.*, 2003; *West et al.*, 2004], it is likely that the spot represents a weather system that has been regenerated time and again, for as long as Jupiter has had its northern magnetic field asymmetry. We do not know how long this is, but given the slow pace of change within the Earth's magnetic field, the Great Cold Spot has existed for thousands of years, and perhaps much longer.

In this context, the Great Cold Spot may represent the first direct evidence of a long‐term vortex confined to a planet's thermosphere within the solar system and opens up the possibility that similar vortices might occur within the thermosphere of all planets with strong auroral activity, at least on the short term, and potentially as quasi‐stable weather systems on longer term. For Jupiter, it shows that the polar thermosphere is a region of complex dynamics and that neutral flows are likely to result in second‐order currents that ultimately feed back into currents that flow out into the surrounding magnetosphere.

## Supporting information



Supporting Information S1

Table S1
