# Supplementary material for: The Great Cold Spot in Jupiter's upper atmosphere
Source: Geophys Res Lett. 2017 Apr 10;44(7):3000–8. doi: 10.1002/2016GL071956 (PMC5439487; doi:10.1002/2016GL071956)
Supplement: Supplementary file 1 — Supporting Information S1 [file GRL-44-3000-s001.docx]

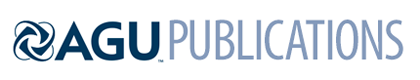


*Geophysical Research Letters*

Supporting Information for

**The Great Cold Spot in Jupiter’s upper atmosphere**

Tom S. Stallard1*, Henrik Melin1, Steve Miller2, Luke Moore3, James O’Donoghue3, John E. P. Connerney4, Takehiko Satoh5, Robert A. West6, Jeffrey P Thayer7, Vicki W. Hsu7, Rosie E. Johnson1

1Department of Physics and Astronomy, University of Leicester, University Road, Leicester LE1 7RH, U.K.

2Department of Physics and Astronomy, University College London, Gower Street, London WC1E 6BT, U.K.

3Center for Space Physics, Boston University, 725 Commonwealth Avenue, Room 506, Boston, MA 02215, USA

4Goddard Space Flight Center, NASA, Mail Code: 695, Greenbelt , MD 20771

5Institute of Space and Astronautical Science, JAXA, Yoshinodai 3-1-1, Chuo-ku, Sagamihara, Kanagawa, 252-5210, Japan

6Jet Propulsion Laboratory, California Institute of Technology, M/S 183-501, 4800 Oak Grove Drive, Pasadena, California 91109-8099, USA

7Aerospace Engineering Sciences, University of Colorado at Boulder, Boulder, CO 80309-0431

*Correspondence to: tss8@leicester.ac.uk

**Contents of this file**

Text S1

Figures S1 to S2

**Additional Supporting Information (Files uploaded separately)**

Captions for Tables S1

**Introduction**

Here, we provide a detailed description of the processing used to convert the IRTF/NSFCam images into latitude vs longitude maps of average emission.

Text S1: Processing of the NSFCam images

The images used in this study come from an extensive set of observations made by Jack Connerney and Takehiko Satoh over the period 1995-2000. This data was recently re-processed by M. Lystrup and B. Bonfond for the ‘Magnetospheres of the Outer Planets Infrared Data Archive’. This data archive provides individual images that consist of sky subtracted, shift-added and flat-fielded images. These images consist of a wide range of different wavelength settings, mostly focused on wavelengths with H3+ emission. We use the five most common settings. Earlier observations use the NSFCam CVF centered on three different wavelengths, 3.420, 3.430 and 3.460 micron, with a spectral resolution of ~50. However, during the period of these observations two very narrow fixed filters we installed, with a central wavelength of 3.4265 and 3.542 micron and a spectral resolution of 200, so that most images from later years come from these two filters. The overall response of these five filters is very similar, as is shown in Figure S1, with bright auroral emission within all the filters. Sunlight is absorbed by broad methane absorption lines in these wavelengths, so that the H3+ emission from the upper atmosphere is isolated; the only significant difference between wavelengths is a weak signal measured in the equatorial region within the 3.542 micron filter, perhaps from a gap in methane absorption close to 3.52 micron; this leads to a slight band non-H3+ emission at the equator.

The precise filter distributions for each of these settings is not currently available, so we are unable to produce precise values for H3+ line intensity. Instead, here, we rely upon our empirical measurements showing that each wavelength band is broadly similar, largely measuring a similar set of H3+ emission lines at a similar range of discrete energy steps, and upon the large number of images investigated effectively smoothing out any wavelength dependent differences.

One common feature of all the images within Figure S1 is the apparent oval of brightness in the equatorial region. This is a ghosting within the imager itself, where regions of bright light are reflected and rotated within the instrument to produce a false signal. This is clearer in Figure S2a, where both reflected sunlight from Io and the bright auroral emission result in separate ‘ghosts’. A nuanced correction for the ghosting was attempted, but the variability of the ghosting positioning from image to image was too large, so instead, we calculate a region in which this ghosting should occur and eliminate this from our study. In order to remove the region effected by this ghosting, we have empirically calculated the offsets needed to exclude the emission: we smooth the original image by a boxcar 20 pixel smoothing in both x and y; we rotate the image by 180 degrees; images from 1995-1997 are not resized, but those from 1998-2000 are increased in size by 1.166; we then offset the image by [-20,-20], [-20,-20], [-15,-15], [-55,-35], [-55,-50], [-55,-45] in [x,y] for the years between 1995 and 2000 respectively. This results in the image shown in Figure S2b. Using this, we then define a ‘ghosting boundary’, where this image is brighter than 17.5. This area is then set to zero within the original image, as show in Figure S2c. We also identify the center of any bright moon within the image and remove any emission from +/- 40 pixels in both x and y, in order to ensure the bright emission from the moons does not contaminate our images.

Once these distortions to the image have been accounted for, we calculate the center of the planet. Initially, we used a limb-finding technique to calculate the planetary center, but after multiple iterations, this proved to have very poor accuracy, and so ultimately, we decided to fit the center of the planet by-eye. To do this, we calculated the shape and size of Jupiter’s limb and overlain this limb on top of the planetary image, clicking to select a suitable center of planet. The position of the calculated planetary limb was compared with the planetary disk, allowing us to fit the planet’s limb by-eye. We estimate that this resulted in a positional accuracy of <0.5”. An example of the result of this fitting is shown in Figure 3a. Using the center of the planet, accounting for Jupiter’s angular diameter, polar flattening and sub-Earth latitude, it is then possible to determine the latitude and longitude for each corner of each pixel within each image that transects the planetary disk. Using the values at the four corners, the position of each pixel is mapped onto to a latitude and longitude grid, which results in a latitude vs longitude map for each individual image, as shown in Figure S3b-c.

In order to ensure that the affects of varying integration time and coadding multiple images, two arrays are produced, one containing the total intensity measured for each pixel and the other containing the total integration time. These are then combined over a single night (in order to produce the images in Figure 3), or co-added across all the nights (to produce Figure 2).

We note that we have not calculated a line-of-sight correction for these images, as the emission we measure is strongest at the limb, while the weak reflected sunlight signal in each image is limb-darkened, so that limb brightening artificially enhances the non-H3+ signal.

**Figure S1:** The five wavelength filters used in this study. Example images for each filter are shown at the top, from left to right: a CVF image centred at 3.420 micron taken on 07 Aug. 1997 at 10:39; a CVF image centred at 3.430 micron taken on 08 July 1996 at 11:45; a CVF image centred at 3.460 micron taken on 29 June 1995 at 08:20; a Connerney filter image centred at 3.4265 micron taken on 19 Dec. 2000 at 10:19; and a Connerney filter image centred at 3.542 micron taken on 19 Dec. 2000 at 09:33. Under this is a normalised theoretical spectra of H3+ at 1000 K (red vertical lines) over-plotted with a standard spectrum of Jupiter (orange line). The regions covered by each filter are shown as grey boxes overlain on this spectrum.

**Figure S2:** Corrections made to the H3+ images before mapping. This image was taken at 07:32 on 11 Oct 99, using the 3.4265 micron filter, shown smoothed to help identify features in panel a). In some images, as here, a bright moon crosses the disk of the planet (in this case Io). The moon’s position is fitted by eye (black cross) and a bounding box around the moon is identified (dashed box). Each image also has a certain amount of ghosting of bright features within the image, here the aurora and Io, shown by the arrows. This ghosting is modelled, as shown in panel b), by smoothing, rotating, resizing and shifting the original image in order to calculate where the ghosting will occur. Areas of this image above a threshold brightness are identified (dashed regions). These regions, along with the region around bright moons, are then excluded, as shown in panel c).

**Figure S3:** Mapping the H3+ images. The center of the planet (white cross) is calculated by fitting the calculated position of the planetary limb to the apparent image within the image by-eye, shown in panel a). Using this, the latitude and longitude of the corner of each pixel in the image that transects the planet is calculated (dotted lines show contours of 30 degrees of both latitude and longitude). Each of these planetary pixels is then translated from the image into a two dimensional map of latitude and longitude, which, when repeated over all planetary pixels results in a cylindrical map of latitude vs longitude, shown in panel b). Using this map, it is then possible to present the data in any preferred mapping projection – within the paper, we provide a view of the auroral region using a polar projection like that shown in panel c).

**Table S1.** The nights of observation used in this study, along with details of the observation: The total integration time on Jupiter across all five filters, the observational period in UT, the total number of images used, the calculated seeing on the night, the angle between Earth, Jupiter and the Sun, the Central Meridian Longitude range observed on the night, the Sub-Earth latitude, Jupiter’s equatorial angular diameter, and the instrument filters used on each night (A: 3.420 micron, B: 3.430 micron, C: 3.460 micron, D: 3.4265 micron and E: 3.542 micron).
